# Revised Manuscript with Corrections: Polyurethane-Based Conductive Composites: From Synthesis to Applications

**DOI:** 10.3390/ijms23041938

**Published:** 2022-02-09

**Authors:** Soon-Mo Choi, Eun-Joo Shin, Sun-Mi Zo, Kummara-Madhusudana Rao, Yong-Joo Seok, So-Yeon Won, Sung-Soo Han

**Affiliations:** 1 Research Institute of Cell Culture, School of Chemical Engineering, Yeung-Nam University, 280 Daehak-ro, Gyeongsan 38541, Korea; smchoi@ynu.ac.kr; 2 Department of Organic Materials and Polymer Engineering, Dong-A University, 37 Nakdong-daero 550beon-gil, Saha-gu, Busan 49315, Korea; sejoo6313@dau.ac.kr; 3 School of Chemical Engineering, Yeung-Nam University, 280 Daehak-ro, Gyeongsan 38541, Korea; sunmizo@ynu.ac.kr (S.-M.Z.); msraochem@yu.ac.kr (K.-M.R.); yjseok@yu.ac.kr (Y.-J.S.); soyeon1005@ynu.ac.kr (S.-Y.W.)

**Keywords:** polyurethane, nanocomposite, conducting filler, conductive, electrical

## Abstract

The purpose of this review article is to outline the extended applications of polyurethane (PU)-based nanocomposites incorporated with conductive polymeric particles as well as to condense an outline on the chemistry and fabrication of polyurethanes (PUs). Additionally, we discuss related research trends of PU-based conducting materials for EMI shielding, sensors, coating, films, and foams, in particular those from the past 10 years. PU is generally an electrical insulator and behaves as a dielectric material. The electrical conductivity of PU is imparted by the addition of metal nanoparticles, and increases with the enhancing aspect ratio and ordering in structure, as happens in the case of conducting polymer fibrils or reduced graphene oxide (rGO). Nanocomposites with good electrical conductivity exhibit noticeable changes based on the remarkable electric properties of nanomaterials such as graphene, RGO, and multi-walled carbon nanotubes (MWCNTs). Recently, conducting polymers, including PANI, PPY, PTh, and their derivatives, have been popularly engaged as incorporated fillers into PU substrates. This review also discusses additional challenges and future-oriented perspectives combined with here-and-now practicableness.

## 1. Introduction

Among various insulating polymers matrices, such as epoxies, alkyds, polyacrylates, polyurethanes (PUs), and others, PUs in particular consist of isocyanates of hard segments and polyols of soft segments, and are one of the widest applicable polymers through tailoring polymer structures. Therefore, they have various polymer properties. PUs were first synthesized by German professor Bayer and his colleagues [1]. They have outstanding properties, including excellent elasticity, elongation, high impact, tensile strength, high abrasion resistance, good weathering resistance, excellent gloss, color retention, and corrosion resistance properties [2]. The aforementioned properties allow them to find applications in a variety of industries, including leather [3], foams [4,5,6], furniture, fibers [7,8,9], elastomers [10], adhesives [11], paints [12,13], coatings [14], sensors [7], electronic components [15], biomedical [16,17], and others [18,19]. In spite of versatile applications, the use of standalone PU often cannot ensure that sufficient thermal and mechanical performances are attained.

For these reasons, it is inevitable to supplement character by blending and composite type in PUs. The effect of introducing conducting polymers into insulating matrices, such as PU, is already well-known. This effect is associated with improved processability, stability, and solubility and better thermal, mechanical, electrical, and optical properties. These composites have infinite potential as functional materials for various industries, thereby motivating researchers. However, interestingly, only seven review articles related to conducting polymer in PU matrices have been published since 1993 [2,20,21,22,23,24,25,26]. Nevertheless, their steady research progress has been reported, which has brought about extended applications of PUs.

Interest in the current technology has highlighted nanocomposites composed of conducting polymers and a non-conducting PU matrix. The emerging applications of the nanocomposites involve electronic devices, actuators, batteries, chemical/biological sensors, etc. The target of conductive polymers is to fabricate polymeric materials with excellent mechanical properties, good processability, and high conductivity. The objective is the preparation of conducting polymeric materials with good mechanical properties and processability associated with high conductivity or electrochromism [27,28]. The incorporation of conducting polymer in a PU matrix is performed covalently or non-covalently, leading to improved characteristics such as conductivity.

This review article aims to review not only the increased applications of the nanocomposites with conducting polymers in conducting but also summarize an outline on chemistry, fabrication, and applications such as electromagnetic interference (EMI) shielding, sensors, coating, and films and foams. Furthermore, we provide interesting challenges and future-oriented perspectives combined with here-and-now practicableness.

## 2. Synthesis of Polyurethanes (PUs)

PUs are the only class of plastic, i.e., thermosetting or thermoplastic, rigid or flexible, solid or open cellular type, with extensive alterations in a property. The sort, site, and structure of both isocyanate and -ol determine not only the progress of PUs’ forming reactions, but also their properties and final applications [29]. They are formed via a chemical reaction which is in step-growth or condensation polymerization, between an isocyanate (di or poly) and -ol (di or poly) to form a prepolymer. Segmented PU comprises a soft segment with a low T_g_ and a hard segment of isocyanate and chain-extender. The prepolymers react with the chain extender for increasing a molecular weight and building a linear block copolymer with alternating blocks of the hard segment and soft segment [30]. The main reaction in PU synthesizing is the formation of the carbamate or urethane bond occurred as the isocyanate reacts with an alcohol and the urea bond generated as the isocyanate reacts with an amine. The reaction can be affected by some parameters, including steric hindrance, enabling the reaction or adjacent electron-withdrawing groups to be slowed down, in turn allowing an increase in the degree of reactions [31].

The polyols employed in PU involve polyesters, polyethers, polycarbonates, and combinations of these polyols in the formation of diblocks and triblocks. The main isocyanates qualified for generating biomedical PU are aromatic and aliphatic. The polyols allow the resultant PUs to possess outstanding tensile properties with high Tm, and the isocyanates enable them to be degradable in order to avoid any toxic issue. Diols or diamines are generally used as chain extenders, which react with isocyanates to build PU’s Mw and increase the length of block in the hard segment. The raw materials used in PU synthesis are summarized in Table 1, Table 2 and Table 3.

Bio-based PU is mainly prepared by a pre-polymerization process, of which hydroxyl or isocyanate-terminated prepolymers are fabricated by the reaction of the desired macroglycol with diisocyanate. Then, a chain extender and a branch-generating moiety are supplemented to the prepolymer to acquire a polymer with a high Mw. The PU is mainly accompanied with a solvent, such as xylen, tetrahydrofuran (THF), dimethylformamide (DMF), dimethylacetamide (DMAc), or dimethylsulfoxide (DMSO). When a branch-generating moiety is added to the prepolymer, high-branched PU (HPU) is obtained, which creates a gel with solvent.

The PU could be modified according to the demands of their applications alternated with bio-based materials; as such, it is necessary to obtain the desired polyols utilized as chain-extenders for the manufacture of bio-based PU. This involves modifications, including hydroxylation of vegetable oils, amidation of tannic acid, epoxidation of unsaturated natural products, modification of starch with epichlorohydrin and bisphenol A, glycerolysis of citric acid, etc. [49,50].

## 3. Diverse Types of Conductive PU-Based Nanocomposites

Some of the main characteristics in outstanding conducting polymers such as graphene oxide (GO) and carbon nanotubes (CNT) include electrical superconductivity, excellent stability, and a simple synthesis. Recently, conducting polymers, including polyaniline (PANI), polypyrrole (PPy), polythiophene (PTh), and their derivatives, are popularly engaged as incorporated fillers into PU matrices [2,51,52,53,54,55,56] due to their features, such as high charge to surface ratio, high efficiency, light weight, and low cost [57,58]. Several research groups have reported that a small amount of the conducting polymer, as fillers, is dispersed in PU matrices. Then, it allows for the fabrication of conductive nanocomposites applicable for electronic devices such as sensors [59,60], actuators [61], membranes [62,63], coatings [14], and others [15,16,17,18,19].

**Figure 1:**
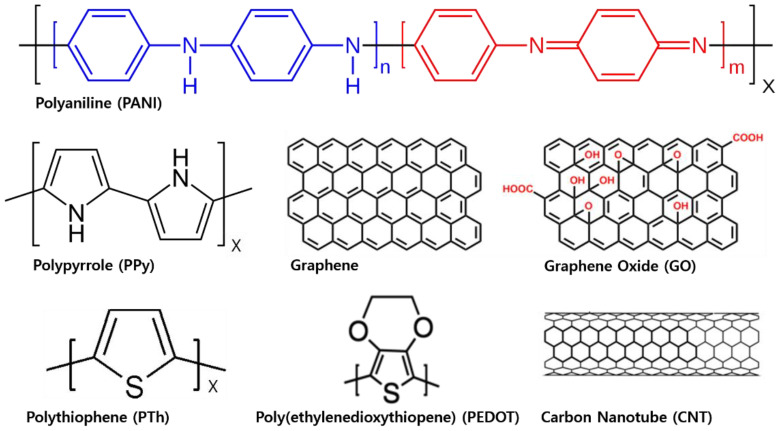
Structures of commonly used conducting polymer as fillers in PU matrices (n + m = 1, x = degree of polymerization).

The conducting polymer fillers involve polyorthotoluidine (POT), polyaniline (PANI), polypyrrole (PPy), polythiophene (PTh), and poly (ethylenedioxythiophene) (PEDOT). The structure of these conducting polymers is shown in Figure 1. Despite common use in a commercial area, there are still unresolved issues in diverse fields such as poor processability, solubility, and mechanical properties. Most researchers have reported several approaches in forming various manners to prepare blends and nanocomposites in their synthesis to overcome the mentioned issues. The properties of nanocomposites primarily depend on the disposition of conducting polymer dispersion in the PU matrices and the interaction of conducting polymer fillers and PU matrices [59].

### 3.1. Polyaniline (PANI)/PUs Composites

**Figure 2:**
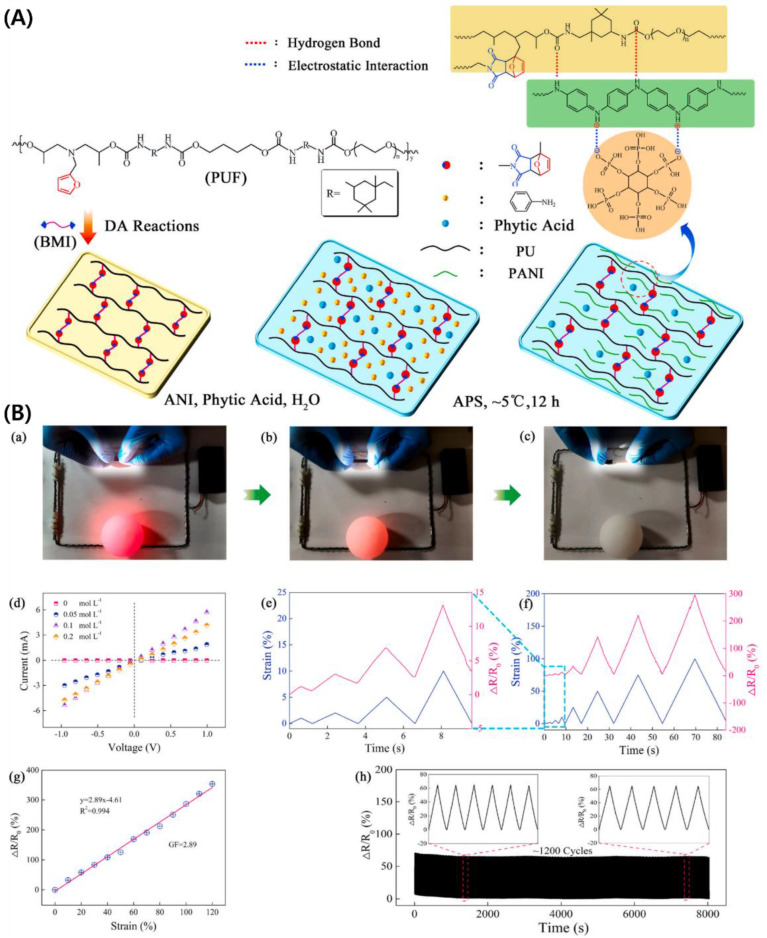
(**A**) Preparation of dually interpenetrating and synergistic network and (**B**) Demonstration of the conductivity of the interpenetrating PANI/PU hydrogels by lighting the LED bulb (**a**) the original sample, (**b**) stretched to a strain about 350%, and (**c**) fracture at strain exceeding 500%. (**d**) C-V curves of PANI/PU hydrogel prepared using different feed concentrations of aniline. (**e**,**f**) relative resistance variation during the successively stretching-releasing from 1% to 100% maximum strain. (**g**) relative resistance variation of the PANI/PU hydrogel as a function of applied strain. (**h**) relative resistance variation during the repeated stretching-releasing deformation for ~1200 cycles at 25% maximum strain. Reprinted with permission from Ref. [64]. Copyright 2021 Elsevier.

PANI is a broadly studied intrinsically conducting polymer using dopants, which is employed to project soft films for flexible strain sensors. Wang et al. fabricated a novel stretchable, sensitive, self-healing, and recyclable PANI/PU hydrogel sensor through in-situ polymerization of aniline, generating a double network [64]. The prepared hydrogel showed 1.1 MPa of strength, 0.3 MPa of Young’s modulus, and 500% of elongation at break, which were skin-like mechanical properties, as shown in Figure 2. The hydrogel sensor also has an outstanding conductivity of 7.87 S m^−1^ and a sensitivity of 2.89.

Athawale et al. prepared PU films modified with nanocomposites of PANI-zinc oxide for biofouling mitigation in 2019 [65]. It was found that this was effective in reducing the adhesion of marine bacteria due to the inherent conductivity of PANI and the photocatalytic effect of zinc oxide (ZnO). The mechanical properties and bioaffinity of PANI were anticipated to be upgraded by incorporating small amounts of nano ZnO. In addition, they could obtain enhanced environmental stability associated with long-term applications and minimize the leaching rate of ZnO from the composites. In 2013, Qin et al. reported that conductive PANI/PU fibers with elasticity were fabricated through in-situ chemical oxidative polymerization of PANI on the surface of PU fibers [66]. The conductivity of the composites depended on PANI contents in which it was decreased for higher PANI contents with a maximum value of 10^−2^ (Ω∙cm)^−1^ for 6–7 wt.% PANI. The piezoresistivity of the resultant composites was shown to have a much higher strain range by up to 400%.

### 3.2. Polypyrrole (PPy)PUs Composites

**Figure 3:**
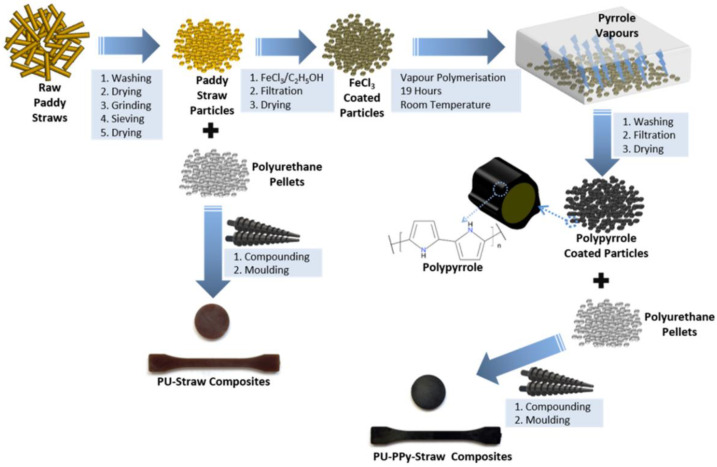
Schematic outline of preparation of PPy/PUs composites coated paddy straw particles and uncoated paddy straw particles. Reprinted with permission from Ref. [67]. Copyright 2020 Elsevier.

Among various conducting polymer fillers, PPy has shown excellent electrical properties and focused on promising materials in view of the electrical composite perspective. Tyagi et al. developed percolative polyurethane-polypyrrole-straw composites with enhanced dielectric constant and mechanical strength via vapor phase polymerization, as shown in Figure 3 [67]. It was reported that the fabricated composites showed 26% increased tensile properties and 133% enhanced dielectric constant, low dissipation factor, and 51.3% decreased water uptake, accordingly having the potential for a wide applications including sensors, actuation, energy generation, and microelectronics.

**Figure 4:**
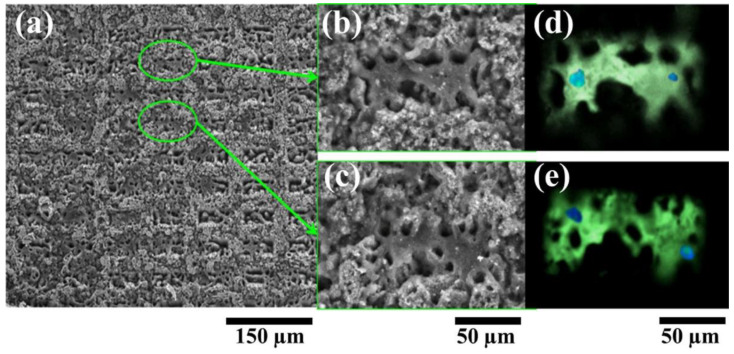
Scanning Electron Micrographs of MG-63 cells growing on the micropatterned PPy/PU substrates (**a**) with insets showing Scanning Electron Micrographs of cells growing inside the rectangular patterns (**b**,**c**) and their corresponding fluorescence microscopy images (**d**,**e**). Reprinted with permission from Ref. [70]. Copyright 2018 Elsevier.

**Figure 5:**
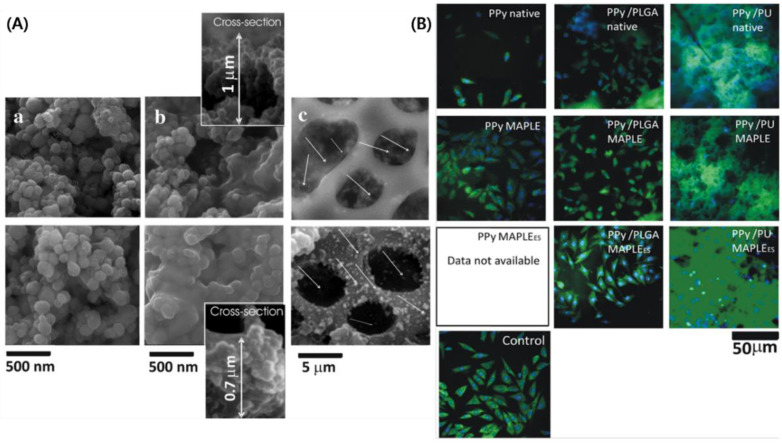
(**A**) SEM micrographs of the PPy-based layers: (upper panel) native; (lower panel) deposited by MAPLE. (**a**) PPy; (**b**) PPy/PLGA; (**c**) PPy/PU (the white arrows point toward the PPy nanograins). The insets from (**b**) display the PPy/PLGA layers viewed in cross-section, and (**B**) Morphological appearance of the MG63 osteoblast-like cells stained with AO (green)/HO (blue) after 1 day of cell culture on PPy, PPy/PLGA and PPy/PU layers: (upper) native; (middle) deposited by MAPLE; (lower) deposited by MAPLE and electrically stimulated. Reprinted with permission from Ref. [71]. Copyright 2015 Elsevier.

Merlini and Barra et al. investigated comparative studies about the effect of montmorillonite/PPy and PPy content on the properties of PU solution, morphology, and mechanical/electrical properties. It was found that the morphology, fiber diameter, properties, and electrical conductivity of the electrospun composites were affected by the collector and filler type [68]. Further, an approach was demonstrated for configuring the neural prostheses, leading to dramatic improvements in adherence, proliferation, and differentiation of both PC12 cells and Schwann cells by Kim et al. [69]. They conducted the study of PPy-coated aligned (inner) and random (outer) layered nanofibrous composites for two-layered nerve guidance conduit (NGC) to guide neuronal extension and regeneration for enhancing tear resistivity. They examined the potential for efficient application of engineered NGC. In that same year, Paun et al. demonstrated the efficiency of micropatterned PPy/PU composites prepared through dispersing PPy nanograins within a mechanically resistant PU matrix to improve the osteogenesis in osteoblast-like cells [70]. The coated PPy/PU layer was micropatterned with 3D geometries by laser, and then the composites were coated by Matrix-Assisted Pulsed Laser Evaporation (MAPLE) to restore their chemical and electrical activity integrity. They aimed to modulate cellular behavior through simultaneous morphological signals and electrical stimulation. The fluorescence staining of Figure 4 notes about two fully developed cells per rectangular pattern. They could control the expression of the single cell to about 50 × 50 μm^2^. One of their studies, related to the PPy/PU composite for bone regeneration, was also conducted in 2015, showing the effect of electrically conductive layers of the composites on bone regeneration [71]. In this study, they also successfully used the MAPLE technique to fabricate biocompatible, electrically conductive PPy/PU composite layers. In addition, it was found that electrical stimulation of 200 μA currents passing through the composite layers for 4 h increased osteogenesis in the cells. The SEM image in Figure 5 confirms the morphology and size of PPy nanograins and offers their incorporation in PU matrices.

**Figure 6:**
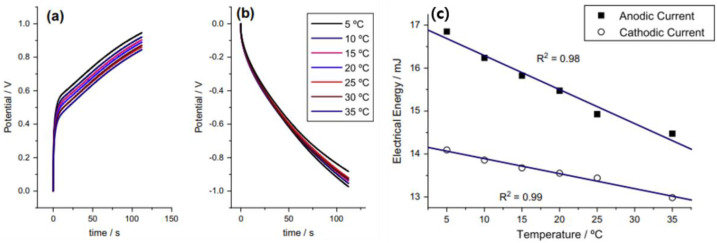
Chronopotentiograms obtained at different temperatures, indicated in the figure, when (**a**) +0.2 mA and (**b**) 0.2 mA were applied to a PU/PPy micro-fibrous mat for 116 s in 1 M NaCl aqueous solution, (**c**) Variation of consumed electrical energy during reaction at different temperatures for anodic and cathodic processes of PU/PPy mat. Reprinted with permission from Ref. [72]. Copyright 2014 Elsevier.

In addition, polymeric nano/micro-fibers as phases have been employed for their chemical coating with polymeric materials. Coated fibers have been used to develop artificial muscles [72,73], supercapacitors and batteries [74,75], and sensing applications [76] due to the large surface area, high electrochemical reactivity, and short path length for the diffusion of ions from the solution compared with the direct employment of conducting polymer films [73]. Otero et al. developed a chemically generated PPy on a PU microfibrous matrix, sensing the surrounding conditions [72]. It was reported that the PPy-incorporated PU mat was prepared using a PU electrospun mat as a template for in situ pyrrole polymerization. The fabricated composite mat displayed a good electroactivity and high porosity and specific surface area. The results showed promise in sensing actuators tools and robots based on conducting polymers. Owing to the increasing of the temperature, the reaction has been made easier because of Arrhenius temperature dependence, faster and longer conformation movements of the chains, and greater diffusion coefficients. As shown in Figure 6, they investigated the sensing capability of the fabricated fibrous matrix electrode by recording chronopotentiograms at diverse temperatures in which chare was kept constant. It was shown that the consumption of electrical energy, while the reaction was occurring for both oxidation and reduction, was a linear function of the temperature.

### 3.3. Poly(Ortho-Toluidine)(POT)PUs Composites

One of the other electrical features of replaced polyaniline, such as poly(ortho-toluidine) (POT), for diverse applications has inflamed researchers base passion, especially facing the development of electrochromic devices. The performance of POT nanoparticles dispersed in PU matrices through a solution blending technique was reported in 2014 [64]. The minimal dispersion (0.25–1.0 wt.%) of POT in castro oil polyurethane (COPU) considerably increases the thermal stability, physicomechanical properties, and corrosion resistance performance of these coatings. The potentiodynamic and electrochemical impedance spectroscopy (EIS) measurements exhibited that the POT/COPU-coated MS efficaciously offers protection via a barrier mechanism in anticipation of acid and salt medium to the mild steel. The salt spray test also showed a similar behavior of coatings to that of the acid environment. The POT/COPU coatings have indicated a greater corrosion protective performance than that of the COPU coatings in acid and saline environments.

**Figure 7:**
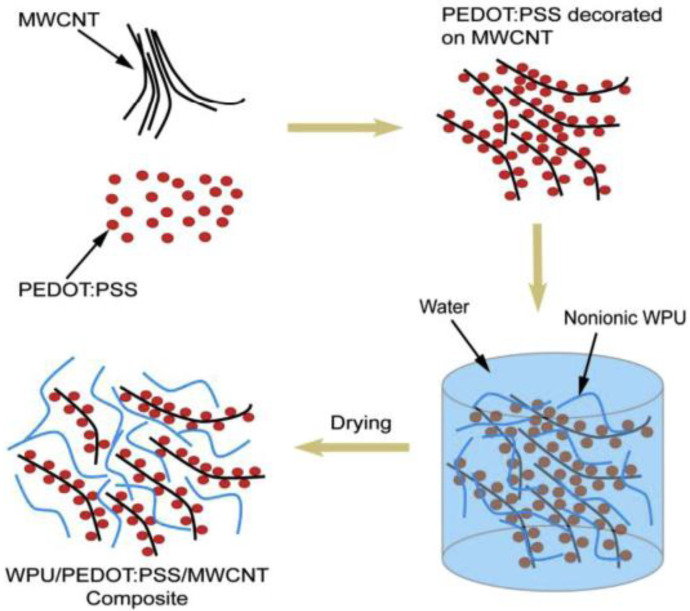
Schematic diagram of the film formation process of nonionic WPU/PEDOT:PSS/MWCNT composite. Reprinted with permission from Ref. [77]. Copyright 2016 Elsevier.

Hu and Wu reported that an eco-friendly thermoelectric composite made of WPU, MWCNT, and PEDOT:PSS was developed for textile yarn coating applications, as shown in Figure 7 [77]. The waterborne polyurethane (WPU) solution they synthesized using PEG, IPDI, and BDO was added into the MWCNT/PEDOT:PSS dispersions, stirred, and sonicated. Then, the mixture was poured into a Teflon mold and dried. After that, the characterization of fabricated composite films displayed enhanced ratios of MWCNT to PEDOT:PSS, the incorporation of DMSO doped highly conductive PH1000, and higher concentration of MWCNT could increase the power factors of the composite. The optimal formulation was indicated at the sample with 20 wt.% MWCNT, 1:4 ratios of MWCNT to PH1000, and 5 wt.% DMSO doping, with having an electrical conductivity of ~13,826 S/m, Seebeck coefficient of ~10 mV/K, and power factor of ~1.41 mW m^−1^ K^−2^ at room temperature. Compared with organic solvent-based polymers, this fabricated waterborne composite showed satisfactory thermoelectric performance and good processability. The application in textile yarn coating was further demonstrated on polyester and cotton yarns, respectively. The results exhibited that the prepared composite could be successfully coated on textile yarns, and the polyester filament was more suitable for coating substrate than staple cotton yarn. Those coated yarns are able to be treated as thermoelectric legs in the future design of fabric thermoelectric generators (TEG). The proposed fabric TEG concept is emerging to overcome the obstacles of the difficulty of wearable flexible film TEG.

### 3.4. Poly(ethylenedioxythiophene)(PEDOT)/PUs Composites

**Figure 8:**
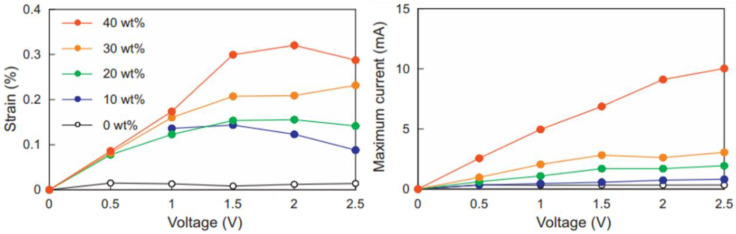
Voltage dependence of strain and maximum current of IL/PU/PEDOT:PSS composite actuators (20 mm long, 5 mm wide, and 130 m thick) with various IL contents. Reprinted with permission from Ref. [78]. Copyright 2014 Elsevier.

Uniform and transparent ILPU gels, consisting of PU elastomer and ionic liquid (IL), were synthesized, and then their mechanical and electrical properties were investigated as shown in Figure 8. It was indicated that IL/PU/PEDOT:PSS composites, which were developed through sandwiching the IL/PU gel between two PEDOT:PSS films as flexible electrodes, showed rapid and intensive bending to anode under the electric field, where the bending displacement of 3.8 mm was obtained at IL 40 wt.%, 2 V, appropriable for the strain of 0.32%. Accordingly, the fabricated electrical composite actuator has potential at frequencies higher than 10 Hz [78]. In another report, T. Chen et al. synthesized PEDOT:PSS noncovalent functionalized graphene–polyurethane dielectric elastomer composites with ultrahigh relative permittivity (350 at 1 kHz), low dielectric loss (0.2 at 1 kHz), low loss modulus (200 Mpa), and low loss tangent(0.4), which are beneficial for the formation of microcapacitors in the matrix and suppressing the leakage current. This result was reported as a promising material for micro-actuator electromechanical applications.

### 3.5. Other Conducting Polymer/PU Composites

**Figure 9:**
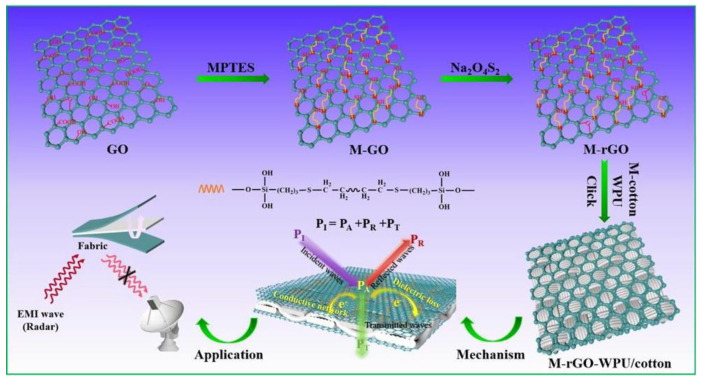
Schematic illustration for the formation of the M-rGO-WPU/cotton fabric and their application for EMI shielding. Reprinted with permission from Ref. [82]. Copyright 2019 Elsevier.

Nowadays, graphene or reduced graphene oxide (rGO), a one-atom-thick two-dimensional carbon lattice, has been widely discussed as an emerging alternative of multifunctional EMI shielding materials due to its surpassing electrical conductivity of 6000 S∙cm^−1^ and thermal conductivity of 5000 W∙m^−1^∙K^−1^ [79,80,81]. Nonetheless, many researchers have been studying to achieve high-performance conductive polymer-reinforced polymer matrix composites. Solving this task, the introduction of rGO fillers has been usually employed for improving compatibility within or on a polymer matrix. For instance, Wang et al. developed through a two-stage bath produce shown in Figure 9 [82]. Briefly, the synthesized WPU with 1, 2 and 3 wt.% of the as-prepared M-rGO (GO modified with 3-mercaptopropyltriethoxysilane (MPTES)) and rGO were prepared and stirred with a mechanical stirrer (300 rpm) for 1 h at room temperature. The prepared cotton fabric was immersed into the mentioned solution and then dried. Next, it was placed under UV irradiation for 30 min. Finally, the M-rGO-WPU/cotton fabric with conductive interconnected network and low M-rGO loadings was fabricated through thiolene click reaction. Owing to the creation of the conductive interconnected network, the resultant fabric exhibited good electrical conductivity, enhanced mechanical properties, higher EMI shielding performance, and capacity of heat transmission. This investigation showed the potential of this material for high-performance EMI shielding applications.

Ding and Xu et al. reported a method for modifying carbon-based materials via forming possible cation-π interactions between the π-electronic surfaces in carbon fillers and 1-aminopropyl-3-methylimidazolium hexafluorophosphate in order to improve the EMI SE of PU composites. The focus of this study is to compare the effect of the geometry of the MWCNTs and graphene nanoplates (GNs), as well as the interaction between Fe_3_O_4_ and carbon fillers on the dielectric properties and electromagnetic interference shielding effectiveness (EMI SE) of WPU composites [83]. WPU-base nanocomposites incorporated with GNs and MWCNTs were developed by polymerizing hybrid emulsions. The carbon fillers displayed excellent dispersion in the PU polymeric phases, as Magnetic Fe_3_O_4_ nanoparticles could offer higher EMI SE of PU/GNs; however they disrupted the EMI SE of PU/MWCNTs because the two-dimensional geometry of the GNs could restrain the strong magnetic attraction between Fe_3_O_4_ and weaken the aggregation of GNs. The results indicated that the distinct morphological discrepancies of GNs and MWCNTs influence the EMI SE and dielectric properties of the PU composites. The tensile strength and the elongation at fracture of neat PU/12 GNs-IL/10Fe_3_O_4_ film could attain 8.9 Mpa and 391.7%, respectively. It eventuated that the PU/GNs-IL/Fe_3_O_4_ composites offer higher SE, further indicating their advantage for an EMI shielding material.

Lee et al. prepared nanocomposites of waterborne polyurethane (WPU) with functionalized graphene sheets (FGS) in situ method. They have also observed a 10^5^ fold increase in the electrical conductivity of WPU after incorporation of factionalized graphene sheets using an in-situ polymerization technique. This result was from that the FGS enhanced the crystallization of the soft segment of WPU evidently [84].

**Figure 10:**
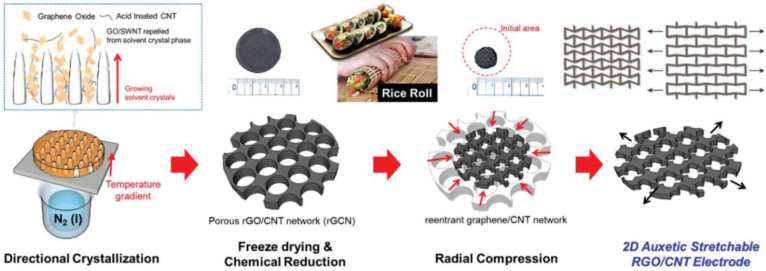
Schematic illustration of the fabrication process of two-dimensional (2D) auxetic reentrant graphene/CNT networks for omnidirectionally stretchable supercapacitor electrodes based on a directional freezing and radial compression process. Reprinted with permission from Ref. [85]. Copyright 2017 RSC Pub.

Son et al. combined a reentrant honeycomb-shaped graphene-CNT structure within a shape-memory polyurethane (SMPU) to accomplish high-performance mechanical properties with graphene-based thermal transport properties [85], as shown in Figure 10. The employed approach related in ice-templated self-assembly and radial compression fabrication caused a directionally porous as well as micro-honeycomb graphene-CNT structure, having continuous conductive paths in vertical and horizontal, highly porous and co-continuous frameworks, and ommi-directional stretchability owing to 2D properties. The facile infiltration of SMPU into the graphene-CNT formed a graphene-CNT framework. The fabricated micro-honeycomb graphene CNT/SMPU composites simultaneously displayed higher electrical/thermal conducting, caused by the interconnected graphene-CNT framework, and excellent tensile shape memory properties because of the arranged carbon/SMPU composite structure.

These nanocomposites exhibit enhanced mechanical properties and thermal stability and showed an excellent electromagnetic wave healable capability. These flexible PU electronics can be employed as flexible conductors and strain sensors to detect the bio-signals of finger bending. S.R. Mustapa et al. prepared Jatropha-oil based polyurethane electrolyte film using lithium perchloride ion (LiClO_4_), replacing conventional petroleum-based polyurethane. The highest conductivity is achieved at 25 wt.% of LiClO_4_ salt content, which is 1.29 × 10^−4^ S/cm at room temperature 30 °C [86].

## 4. Conclusions and Future Perspective

Among diverse insulating polymers phases such as epoxy, alkyd, and polyacrylates, PUs are one of the polymers with a wide range of applications, which have versatile nature due to consisting of both hard and soft segments. Therefore, this allows PUs to be applicable for various industries in leather, coating, elastomers, adhesives, sensors, electronic components, biomedical, and others [3,4,5,6,7,8,9,10,11,12,13,14,15,16,17,18,19]. Recently, it has been shown that the effect between conducting polymers as fillers and insulating PUs as matrices enables increases in their properties involving processability, stability, and solubility and their thermal, mechanical, and electrical properties [76]. Hence, it is critical to introduce structural properties in PU matrices via blending and composite types, thereby enabling good functionality. Although conducting polymers such as polyaniline, polythiophene, and polypyrrole show excellent electrical properties as well as stability, their solubility and mechanical properties result in poor processability. In order to overcome the mentioned limitations, it has been focused on the chemical functionalization of conducting fillers within composites with thermoplastic, highly stretchable polyurethanes being one of the most important classes of functional materials. Moreover, the conducting polymers mentioned above, incorporating polymer matrices with biocompatibility and biodegradability, have more emerging applications in biomedical applications, including tissue engineering, regenerative medicine, drug delivery, and other alternate products. Although in vitro biocompatibility of the conductive fillers has been confirmed, in vivo properties have been still remained a significant uncertainty. Therefore, in vivo studies are additionally demanded to be sure of the nontoxicity of the conducting materials. There is no doubt that a variety of conductive biomaterials with excellent biocompatibility and biodegradability will be extensively developed in the not-too-distant future for biomedical applications.

## Figures and Tables

**Table 1:** Raw materials used in PU synthesis using soft-segment.

Soft-segment	Polyols (-OH)	Polyester segments [29,30,31,32]	Poly (ε-caprolactone)(PCL) diol Poly (D,L,-lactide)(PDLLA) diol Poly (glycolide)(PGA)diol Poly (ethylene adipate) diol	- Rigid PU with good heat and chemical resistance - Susceptible to hydrolysis
		Polyether segments [30,33,34,35]	Poly (ethylene oxide) (PEO) Poly (propylene oxide) (PPO) Poly (tetramethylene oxide) (PTMO) Poly (hexamethylene oxide) (PHMO)	- High moisture permeability and low Tg
		Triblock segments [36,37,38]	PCL-b-PEO-b-PCL diol PCL-b-PPO-b-PCL diol PCL-b-PTMO-b-PCL diol	

**Table 2:** Raw materials used in PU synthesis using hard-segment.

Hard-segment	Isocyanates (R-N=C=O)	Aliphatic [39,40]	1,4-Diisocyanatobutane (BDI) 1,6-Diisocyanatohexane (HDI) Lysine methyl ester diisocyanate (LDI)	- Vinyl terminated isocyanate, which provides site of crosslinking
		Cycloaliphatic [41,42]	Dicyclohexylmethane diisocyanate(H12MDI) Isophorone diisocyanate (IPDI)	
		Aromatic [43]	Methylene diphenyl diisocyanate (MDI)	- Negative charge, more reactive, to produce rigid PU, lower oxidatives & ultraviolet stabilities
	Chain extender	Di/poly-hydroxyl [44]	1,4-Butane diol Cyclohexane dimetnanol	- Require organometallic catalysts
		Hydroxyl amine [45]	Diethanol amine (DEA)	
		Diamine [46]	Ethylene diamine (ED) 1,4-Butanediamine(putrescine)	- Increase bridging with biuret linkages

**Table 3:** Raw materials used in PU synthesis using additives.

Additives	Catalysts	Amines [29]	Diaminobicyclooctane (DABCO)	- By complex formation between amine and isocyanate
		Organometallic compounds [47]	Dibutyltin dilaurate Dibutyltin diacetate	- Toxic and cause disposal problems
		Alkali metal salts of carboxylic acid and phenols [48]	Calcium, magnesium, strontium, barium, salt of hexanoic, octanoic, naphthenic, linolenic acid	

## Data Availability

Not applicable.
